# Letter to the editor: Early adversity and inflammation at midlife: the moderating role of internalizing psychopathology

**DOI:** 10.1017/S0033291725101554

**Published:** 2025-09-29

**Authors:** Hisao Toyoshima, Masayoshi Koinuma, Tomohide Akase

**Affiliations:** 1 Pharmacy Management Institute, Graduate School of Business, Japan University of Economics, Tokyo, Japan; 2 Faculty of Pharmaceutical Sciences, Teikyo Heisei University, Nakano City, Tokyo, Japan; 3 Graduate School of Business, Japan University of Economics, Tokyo, Japan

## Impact of early adversity and adult emotional distress on immune function

The cross-sectional design employed in this study limits definitive interpretation of the causal relationship and temporal development between early adversity and adult emotional distress (Michal, Marquardt, Krueger, Arbisi, & Venables, 2025). Specifically, to comprehensively examine the effects of early adversity and adult emotional distress on inflammation, a comparative analysis of four distinct groups would be more informative: (i) no early adversity and no adult emotional distress, (ii) no early adversity but with adult emotional distress, (iii) early adversity but no adult emotional distress, and (iv) both early adversity and adult emotional distress. The present study’s conclusions are drawn primarily from analysis of group (iv) alone, which represents a methodological limitation. Unfortunately, the current sample size and research design make it difficult to substantiate the potential amplifying effect of early adversity on midlife immune changes (Baumeister, Akhtar, Ciufolini, Pariante, & Mondelli, 2015; Elwenspoek et al., 2017). Longitudinal verification through prospective studies is necessary to address this limitation (Baumeister et al., 2015; Elwenspoek et al., 2017).

## Factors influencing adult emotional distress

Beyond early adversity, several other factors contribute to adult emotional distress, including family environment, workplace conditions, genetic predisposition, and social determinants (Brandt, Liu, Heim, Binder, & Belsky, 2022; Hamouche, 2020; Knyazev, Bocharov, Savostyanov, & Proshina, 2023; Yuan, Zhang, Zhou, & Zhou, 2021). It is essential to adjust for these confounding variables before discussing potential relationships between the primary variables of interest.

Based on these considerations, we respectfully request that the authors address the following points:

Conduct comparative analyses across all four groups (i–iv) to clearly demonstrate the contribution of each factor and their synergistic effects. This would add greater reliability to the examination of causal relationships, providing more robust conclusions than those derived from focusing solely on group (iv).

Either include the four aforementioned confounding factors as covariates or perform appropriate stratified or sensitivity analyses to more rigorously evaluate the influence of the primary independent variables: early adversity and adult emotional distress.

We appreciate your attention to these considerations and look forward to the authors’ response.

We read with great interest the articles by Zachary S. Michal, Paul A. Arbisi, Craig A. Marquardt, and Noah C. Venables titled “Early adversity and inflammation at midlife: the moderating role of internalizing psychopathology” (*Psychological Medicine* 54, 4518–4527). This study makes a valuable contribution by elucidating how internalizing psychopathology moderates the impact of early adversity on adult inflammation, thereby deepening our understanding of the interrelationship between mental and physical health. However, we would like to highlight several limitations and challenges that warrant further consideration.
